# Domain-Specific Associations of Physical Activity With Phase Angle and Extracellular Water–to–Total Body Water Ratio in Middle-Aged or Older Adults: Cross-Sectional Study

**DOI:** 10.2196/90158

**Published:** 2026-09-28

**Authors:** Seong-Uk Baek, Jin-Ha Yoon

**Affiliations:** 1 Graduate School, Yonsei University College of Medicine, Seoul, Seoul, Republic of Korea; 2 Department of Preventive Medicine, Yonsei University College of Medicine, 50-1, Yonsei-ro, Seodaemungu, Seoul, 03722, Republic of Korea, 82 010-34118-697; 3 The Institute for Occupational Health, Yonsei University College of Medicine, Seoul, Republic of Korea; 4 Institute for Innovation in Digital Healthcare, Yonsei University Health System, Seoul, Republic of Korea

**Keywords:** bioimpedance analysis, body fluid, exercise, physical activity, epidemiology

## Abstract

**Background:**

The phase angle (PhA) and extracellular water (ECW)–to–total body water (TBW) ratio serve as indicators of cell membrane integrity and body fluid balance in older adults.

**Objective:**

This study examined the domain-specific associations of physical activity (PA) with PhA and the ECW-to-TBW ratio.

**Methods:**

A nationally representative sample of 5211 adults aged 50 years and older was analyzed. Weekly amounts of moderate-to-vigorous PA (MVPA) in leisure-time, occupational, and transport-related domains were assessed using the Global Physical Activity Questionnaire. A bioelectrical impedance analyzer was used to measure PhA (°) and the ECW-to-TBW ratio (%). Linear regression analyses were conducted.

**Results:**

For leisure-time PA, compared with 0 min/week, participating in 150 to 299 min/week and ≥300 min/week of MVPA was associated with 0.08 (95% CI 0.02-0.14) and 0.13 (95% CI 0.06-0.20) degrees higher PhA, respectively. Additionally, compared with 0 min/week, engaging in 150 to 299 min/week and ≥300 min/week of MVPA was associated with –0.11 (95% CI –0.19 to –0.03) and –0.15 (95% CI –0.22 to –0.07) percentage points lower ECW-to-TBW ratio, respectively. For transport-related PA, compared with 0 min/week, engaging in ≥300 min/week of MVPA was negatively associated with the ECW-to-TBW ratio (β=–0.07, 95% CI –0.14 to –0.01). Occupational PA showed no clear associations with PhA or the ECW-to-TBW ratio.

**Conclusions:**

While the observed associations were modest, the associations of PA with both PhA and the ECW-to-TBW ratio differed across PA domains. Specifically, leisure-time PA was positively associated with PhA and inversely associated with the ECW-to-TBW ratio. These findings highlight the potential role of leisure-time PA in improving bioelectrical markers of cellular health and fluid distribution.

## Introduction

Physical activity (PA) is recognized as a major priority in promoting public health in older adults. While the World Health Organization (WHO) recommends that adults participate in ≥150 min/week of moderate-to-vigorous physical activity (MVPA), a concerning proportion of the global population exhibits low levels of PA [1]. For example, a recent study reported that insufficient PA was observed in 31.3% of the global population in 2022, representing a gradual increase over the past decades [1]. In South Korea, sedentary lifestyles remain a significant public health concern. Despite various governmental policies and campaigns to promote PA, 54.5% of adults did not meet the WHO PA guidelines in 2020 [2], a prevalence that has continued to rise over recent decades [3]. Furthermore, older adults in South Korea were significantly less likely to engage in PA, and the decreasing trend was particularly steep among the population aged 50 years and older [3].

The health benefits of PA for older adults are well established. PA is associated with lower risks of overall and cardiovascular disease mortality in older adults [4]. The mechanisms underlying these health benefits can be attributed to multiple factors, including improvements in metabolic profiles, muscle strength and function, and psychological well-being [5]. However, accumulating evidence suggests that the effects of PA vary depending on the domain and context in which it is performed—a phenomenon referred to as the “PA paradox” [6,7]. For instance, studies have shown that reductions in the risks of all-cause mortality and cardiovascular disease are most pronounced for leisure-time physical activity (LTPA) compared with occupational physical activity (OPA) or transport-related physical activity (TRPA) [8-10]. Furthermore, OPA was linked to an elevated risk of mortality [10]. Despite the existing evidence regarding the “PA paradox,” differential associations between PA domains and body composition or fluid status have rarely been investigated.

The phase angle (PhA) and extracellular water (ECW)–to–total body water (TBW) ratio, both determined using bioelectrical impedance analysis (BIA), serve as biomarkers of body fluid distribution and cellular function [11]. While PhA reflects cellular membrane integrity, the ECW-to-TBW ratio primarily reflects electrolyte balance and edema status [11,12]. Adverse health conditions, such as malnutrition, edema, systemic inflammation, and low skeletal muscle mass, are associated with lower PhA and higher ECW-to-TBW ratios [11,13,14]. Moreover, low PhA and high ECW-to-TBW ratios have been linked to increased mortality risk among older adults [15-17]. Therefore, PhA and the ECW-to-TBW ratio are regarded as important biomarkers of health status in recent literature.

PA substantially affects both body fluid distribution and body composition by reducing body fat and improving muscle quality in older adults [18-20]. These physiological adaptations can influence BIA-derived parameters, including PhA and the ECW-to-TBW ratio. Prior studies, for instance, reported a positive association between total weekly PA and PhA, along with a negative association with the ECW-to-TBW ratio [18-20]. However, whether the associations of PA with PhA or the ECW-to-TBW ratio differ across PA domains in older adults has been rarely examined. Specifically, no previous studies have investigated how LTPA, OPA, and TRPA are differentially associated with PhA and the ECW-to-TBW ratio. To address this research gap, this study investigated the associations of LTPA, OPA, and TRPA with PhA and the ECW-to-TBW ratio in a nationwide sample of older adults in South Korea. Specifically, we hypothesized that (1) LTPA and TRPA would be positively associated with PhA and inversely associated with the ECW-to-TBW ratio, and (2) OPA would be inversely associated with PhA and positively associated with the ECW-to-TBW ratio.

## Methods

### Study Population

This study used a cross-sectional design, with the study sample drawn from the Korea National Health and Nutrition Examination Survey (KNHANES). The Korea Disease Control and Prevention Agency (KDCA) administers the KNHANES annually, providing a nationally representative sample of the Korean population. A multistage clustered probability sampling methodology was used to include participants, ensuring representation of the entire Korean population [21]. In this study, datasets from the 2022 and 2023 cycles of the KNHANES were used, during which BIA was performed on participants. Note that, owing to the cross-sectional study design, the temporal relationship between the exposure variables (PA domains) and the outcomes (PhA and the ECW-to-TBW ratio) could not be established, which limits the ability to draw causal inferences from the study findings. A total of 6804 adults aged 50 years and older participated in the 2022 and 2023 survey years of the KNHANES. Individuals meeting any of the following exclusion criteria were excluded: (1) those with pacemakers or implantable cardioverter-defibrillators, (2) pregnant women, (3) individuals with prosthetic limbs, and (4) those who refused or were unable to undergo BIA. In total, 5742 (84.4%) adults participated in BIA. After further excluding participants with missing data, a final sample of 5211 (76.6%) adults was included in this analysis.

### Ethical Considerations

All participants provided written informed consent. The protocols of the 2022 and 2023 KNHANES were approved by the institutional review board of the KDCA (2018-01-03-4C-A, 2022-11-16-R-A).

### Physical Activity

The Korean version of the Global Physical Activity Questionnaire (K-GPAQ) was used to measure the weekly amounts of total PA, LTPA, OPA, and TRPA. The K-GPAQ has demonstrated good reliability and validity for assessing PA among the Korean population [22]. The questionnaire evaluates PA in 3 domains: LTPA, OPA, and TRPA. LTPA was assessed using the following questions: “Do you regularly participate in vigorous-intensity sports, exercise, or leisure activities that cause you to become significantly out of breath or make your heart beat very fast for at least 10 consecutive minutes?” (vigorous PA) and “Do you regularly participate in moderate-intensity sports, exercise, or leisure activities that cause you to become slightly out of breath or make your heart beat somewhat faster for at least 10 consecutive minutes?” (moderate PA). OPA was assessed using the following questions: “Does your job involve vigorous-intensity physical activity that causes you to become significantly out of breath or make your heart beat very fast for at least 10 consecutive minutes?” (vigorous PA) and “Does your job involve moderate-intensity physical activity that causes you to become slightly out of breath or make your heart beat somewhat faster for at least 10 consecutive minutes?” (moderate PA). TRPA was assessed using the following question: “Do you walk or cycle for transportation (eg, commuting to work, shopping, running errands, attending religious services, or going to or from school or private academies) for at least 10 consecutive minutes?” (moderate PA). For each question, the duration of each session and the number of sessions per week were recorded. The weekly amount of MVPA for LTPA, OPA, and TRPA was then calculated using the following equation:


MVPA(per week)=2×Vigorous PA(per week)+Moderate PA(per week)

Total PA was calculated as the sum of MVPA across the 3 domains—LTPA, OPA, and TRPA. Finally, for each PA domain, PA levels were divided into 4 groups: 0, 1‐149, 150‐299, and ≥300 min/week, in accordance with the WHO PA guidelines [23].

### PhA and ECW-to-TBW Ratio

PhA and the ECW-to-TBW ratio were measured using a multifrequency bioelectrical impedance analyzer (InBody 970; Biospace). The InBody 970 has demonstrated good validity for assessing body composition [24]. Moreover, measurements obtained from the InBody 970, including PhA, show high reliability and are comparable with results from other BIA devices [25]. Before the assessment, participants fasted for at least 8 hours. After removing all accessories and digital devices, they stood barefoot on the InBody 970 while holding the hand electrodes. All BIA procedures were supervised by trained health professionals from the KDCA. Whole-body PhA was recorded to the nearest 0.1° at 50 kHz, and ECW, intracellular water (ICW), and TBW were recorded to the nearest 0.1 L. Both PhA (°) and the ECW-to-TBW ratio (%) were analyzed as continuous variables.

### Covariates

The confounders included the following sociodemographic characteristics: sex, age, educational attainment (middle school or below, high school, or college or above), income (lowest, low, high, or highest), marital status (married, unmarried, or others), smoking status (no or yes), alcohol use (no or yes), hypertension (no or yes), diabetes (no or yes), chronic kidney disease (no or yes), and BMI (kg/m^2^). However, because of the lack of available data, several important confounders, including dietary intake, inflammatory markers, and medication use, which may influence body composition and fluid status, could not be included in the analyses. This limitation may have resulted in residual confounding.

### Statistical Analysis

Sample characteristics were summarized according to total PA levels. Linear regression models were applied to evaluate the associations of PA levels with both PhA and the ECW-to-TBW ratio, using β coefficients and 95% CIs. The normality of PhA and the ECW-to-TBW ratio was confirmed through visual inspection of histograms and Q-Q plots. First, the associations of total PA with PhA and the ECW-to-TBW ratio were evaluated. Second, the associations of LTPA, OPA, and TRPA with PhA and the ECW-to-TBW ratio were examined. Both crude and fully adjusted models were fitted. For further analyses, the PA variables were treated as continuous variables in the model. Additionally, the nonlinear associations between total PA and each domain of PA with PhA and ECW-to-TBW were examined using the restricted cubic spline function with 4 knots [26]. Statistical analyses were performed using R (version 4.5.0; R Foundation for Statistical Computing). The *survey* package and its function *svyglm* were used to account for the sampling design of the KNHANES [27].

Compared with the included participants, those with missing values were more likely to be older, have lower levels of education and income, have lower PhA values, and have a higher ECW-to-TBW ratio (Table S1 in Multimedia Appendix 1). To mitigate this selection bias, for sensitivity analyses, multiple imputation was performed to handle missing data using the R package *mice* [28]. Specifically, 20 complete datasets were generated using the multivariate imputation by chained equations method, and estimates from each dataset were combined using Rubin rules.

## Results

Among the 5211 participants, 31.7% (n=1650), 28.7% (n=1496), 19.2% (n=1000), and 20.4% (n=1065) engaged in 0, 1‐149, 150‐299, and ≥300 min/week of MVPA, respectively (Table 1). Participants with higher levels of total PA tended to be younger, have higher education and income levels, and not have hypertension, diabetes, or chronic kidney disease. The mean PhA values were 5.0° (SD 0.7), 5.0° (SD 0.7), 5.1° (SD 0.7), and 5.2° (SD 0.7) for those reporting 0, 1‐149, 150-299, and ≥300 min/week of MVPA, respectively. The corresponding mean ECW-to-TBW ratios were 38.7% (SD 0.8), 38.4% (SD 0.8), 38.5% (0.7), and 38.4% (SD 0.7), respectively.

In the fully adjusted model (Table 2), compared with 0 min/week of MVPA, ≥300 min/week of MVPA was positively associated with PhA (β=0.08, 95% CI 0.04-0.13). Compared with 0 min/week, the ECW-to-TBW ratio was negatively associated with 150‐299 min/week of MVPA (β=–0.08, 95% CI –0.14 to −0.03), and ≥300 min/week of MVPA (β=–0.11, 95% CI –0.15 to –0.06). Therefore, total PA showed a linear positive association with PhA and a linear negative association with the ECW-to-TBW ratio, even after adjustment for covariates.

**Table 1.** Characteristics of the participants.

	Overall (n=5211)	Total PA^a^ level (MVPA^b^ per wk)			
		0 min (n=1650)	1-149 min (n=1496)	150-299 min (n=1000)	≥300 min (n=1065)
Sex, n (%)					
Male	2256 (43.3)	743 (45)	572 (38.2)	411 (41.1)	530 (49.8)
Female	2955 (56.7)	907 (55)	924 (61.8)	589 (58.9)	535 (50.2)
Age (years), mean (SD)	64.5 (8.7)	65.8 (8.9)	65.1 (8.8)	63.2 (8.2)	62.7 (8.4)
Education level, n (%)					
Middle school or below	2111 (40.5)	818 (49.6)	629 (42)	332 (33.2)	332 (31.2)
High school	1770 (34)	531 (32.2)	501 (33.5)	360 (36)	378 (35.5)
College or above	1330 (25.5)	301 (18.2)	366 (24.5)	308 (30.8)	355 (33.3)
Income level, n (%)					
Lowest	1362 (26.1)	516 (31.3)	424 (28.3)	210 (21)	212 (19.9)
Low	1344 (25.8)	423 (25.6)	391 (26.1)	257 (25.7)	273 (25.6)
High	1175 (22.5)	347 (21)	327 (21.9)	256 (25.6)	245 (23)
Highest	1330 (25.5)	364 (22.1)	354 (23.7)	277 (27.7)	335 (31.5)
Marital status, n (%)					
Married	3979 (76.4)	1211 (73.4)	1126 (75.3)	797 (79.7)	845 (79.3)
Unmarried	149 (2.9)	37 (2.2)	47 (3.1)	32 (3.2)	33 (3.1)
Others	1083 (20.8)	402 (24.4)	323 (21.6)	171 (17.1)	187 (17.6)
Smoking, n (%)					
No	4503 (86.4)	1404 (85.1)	1295 (86.6)	880 (88)	924 (86.8)
Yes	708 (13.6)	246 (14.9)	201 (13.4)	120 (12)	141 (13.2)
Alcohol consumption, n (%)					
No	2920 (56)	983 (59.6)	883 (59)	540 (54)	514 (48.3)
Yes	2291 (44)	667 (40.4)	613 (41)	460 (46)	551 (51.7)
Hypertension, n (%)					
No	3176 (60.9)	931 (56.4)	911 (60.9)	665 (66.5)	669 (62.8)
Yes	2035 (39.1)	719 (43.6)	585 (39.1)	335 (33.5)	396 (37.2)
Diabetes, n (%)					
No	4346 (83.4)	1320 (80)	1245 (83.2)	862 (86.2)	919 (86.3)
Yes	865 (16.6)	330 (20)	251 (16.8)	138 (13.8)	146 (13.7)
Chronic kidney disease, n (%)					
No	5049 (96.9)	1604 (97.2)	1446 (96.7)	969 (96.9)	1030 (96.7)
Yes	162 (3.1)	46 (2.8)	50 (3.3)	31 (3.1)	35 (3.3)
BMI (kg/m^2^), mean (SD)	24.1 (3.3)	24.3 (3.4)	24.1 (3.2)	23.9 (3.2)	24.2 (3.3)
Phase angle (°), mean (SD)	5.1 (0.7)	5.0 (0.7)	5.0 (0.7)	5.1 (0.7)	5.2 (0.7)
ECW^c^-to-TBW^d^ ratio (%), mean (SD)	38.6 (0.8)	38.7 (0.8)	38.7 (0.8)	38.5 (0.7)	38.4 (0.7)

a PA: physical activity.

b MVPA: moderate-to-vigorous physical activity.

c ECW: extracellular water.

d TBW: total body water.

**Table 2.** Associations of total physical activity (PA) with the phase angle and extracellular water (ECW)–to–total body water (TBW) ratio.

Total PA (MVPA^a^)	Phase angle (°)		ECW-to-TBW ratio (%)	
	Crude model, β (95% CI)	Adjusted model, β (95% CI)	Crude model, β (95% CI)	Adjusted model, β (95% CI)
0 min/wk	Reference	Reference	Reference	Reference
1-149 min/wk	−0.02 (−0.09 to 0.04)	0.02 (−0.01 to 0.06)	−0.03 (−0.09 to 0.03)	−0.04 (−0.08 to 0.01)
150-299 min/wk	0.09 (0.02 to 0.17)	0.04 (−0.01 to 0.09)	−0.19 (−0.27 to −0.12)	−0.08 (−0.14 to −0.03)
≥300 min/wk	0.23 (0.16 to 0.30)	0.08 (0.04 to 0.13)	−0.28 (−0.35 to −0.21)	−0.11 (−0.15 to −0.06)

a MVPA: moderate-to-vigorous physical activity.

Table 3 presents the associations of the levels of LTPA, OPA, and TRPA with both PhA and the ECW-to-TBW ratio. For LTPA, compared with 0 min/week, engaging in 1-149, 150-299, and ≥300 min/week of MVPA was associated with 0.02 (95% CI –0.03 to 0.06), 0.08 (95% CI 0.02-0.14), and 0.13 (95% CI 0.06-0.20) degrees higher PhA, respectively. In addition, compared with 0 min/week, participating in 1-149, 150-299, and ≥300 min/week of MVPA was associated with –0.06 (95% CI –0.12 to −0.00), –0.11 (95% CI –0.19 to −0.03), and –0.15 (95% CI –0.22 to −0.07) percentage points lower ECW-to-TBW ratio, respectively. For TRPA, compared with 0 min/week, engaging in ≥300 min/week of MVPA was negatively associated with the ECW-to-TBW ratio (β=–0.07, 95% CI –0.14 to –0.01). However, no clear relations were observed between TRPA and the PhA. Finally, OPA showed no significant associations with either PhA or the ECW-to-TBW ratio. Therefore, the associations with both PhA and the ECW-to-TBW ratio were most consistent for LTPA, whereas TRPA was associated only with a lower ECW-to-TBW ratio, and OPA was not significantly associated with either outcome.

**Table 3.** Associations of leisure-time, occupational, and transport-related physical activity with the phase angle and extracellular water (ECW)–to–total body water (TBW)^,^ ratio

	Phase angle (°)		ECW-to-TBW ratio (%)	
	Crude model, β (95% CI)	Adjusted model, β (95% CI)	Crude model, β (95% CI)	Adjusted model, β (95% CI)
0 min/wk	Reference	Reference	Reference	Reference
LTPA^a^ (MVPA)^b^				
1-149 min/wk	0.21 (0.13 to 0.28)	0.02 (−0.03 to 0.06)	−0.28 (−0.35 to −0.22)	−0.06 (−0.12 to –0.00)
150-299 min/wk	0.25 (0.17 to 0.34)	0.08 (0.02 to 0.14)	−0.33 (−0.43 to −0.23)	−0.11 (−0.19 to −0.03)
≥300 min/wk	0.43 (0.33 to 0.53)	0.13 (0.06 to 0.20)	−0.44 (−0.54 to −0.35)	−0.15 (−0.22 to −0.07)
OPA^c^ (MVPA)				
1-149 min/wk	0.13 (−0.00 to 0.26)	0.04 (−0.05 to 0.14)	−0.01 (−0.14 to 0.11)	0.02 (−0.08 to 0.12)
150-299 min/wk	0.02 (−0.20 to 0.24)	−0.09 (−0.21 to 0.03)	0.04 (−0.19 to 0.28)	0.13 (−0.02 to 0.29)
≥300 min/wk	0.31 (0.20 to 0.42)	0.03 (−0.04 to 0.10)	−0.21 (−0.31 to −0.10)	0.05 (−0.03 to 0.14)
TRPA^d^ (MVPA)				
1-149 min/wk	−0.14 (−0.19 to −0.09)	−0.00 (−0.04 to 0.03)	0.07 (0.02 to 0.13)	−0.02 (−0.07 to 0.02)
150-299 min/wk	−0.06 (−0.13 to 0.01)	0.02 (−0.03 to 0.08)	−0.02 (−0.09 to 0.05)	−0.06 (−0.11 to 0.00)
≥300 min/wk	−0.04 (−0.12 to 0.05)	0.03 (−0.03 to 0.08)	−0.02 (−0.10 to 0.07)	−0.07 (−0.14 to −0.01)

a LTPA: leisure-time physical activity.

b MVPA: moderate-to-vigorous physical activity.

c OPA: occupational physical activity.

d TRPA: transport-related physical activity.

Figure 1 shows the nonlinear associations of LTPA, OPA, and TRPA with PhA and the ECW-to-TBW ratio based on restricted cubic spline models. For PhA, a pronounced positive association was observed with LTPA. TRPA showed a weak positive association, whereas OPA exhibited a flat pattern, indicating no clear association with PhA. For the ECW-to-TBW ratio, LTPA demonstrated the strongest inverse association, whereas TRPA was also negatively associated with the ECW-to-TBW ratio. In contrast, OPA showed a positive association up to approximately 100 min/week of MVPA, beyond which the association was no longer apparent.

Sensitivity analyses based on the imputed datasets confirmed that total PA was directly associated with PhA and inversely associated with the ECW-to-TBW ratio (Table S2 in Multimedia Appendix 1). The slight differences between the estimates in Table 2 and Table S2 in Multimedia Appendix 1 reflect the differences in analytic approaches between complete-case analysis and multiple imputation, while the estimates are comparable. Moreover, the associations of LTPA, OPA, and TRPA with both PhA and the ECW-to-TBW ratio remained consistent in the sensitivity analyses (Table S3 in Multimedia Appendix 1). When PA was analyzed as a continuous variable (Table S4 in Multimedia Appendix 1), each 150-min/week increment in MVPA from LTPA, OPA, and TRPA was associated with changes in PhA (**°**) of 0.03 (95% CI 0.01-0.05), 0.00 (95% CI −0.01 to 0.01), and 0.01 (95% CI −0.01 to 0.02), respectively. The corresponding changes in the ECW-to-TBW ratio (%) were −0.03 (95% CI −0.05 to −0.01), 0.01 (95% CI −0.01 to 0.02), and −0.02 (95% CI −0.04 to −0.01), respectively, after adjustment for covariates (model 2).

**Figure 1.**
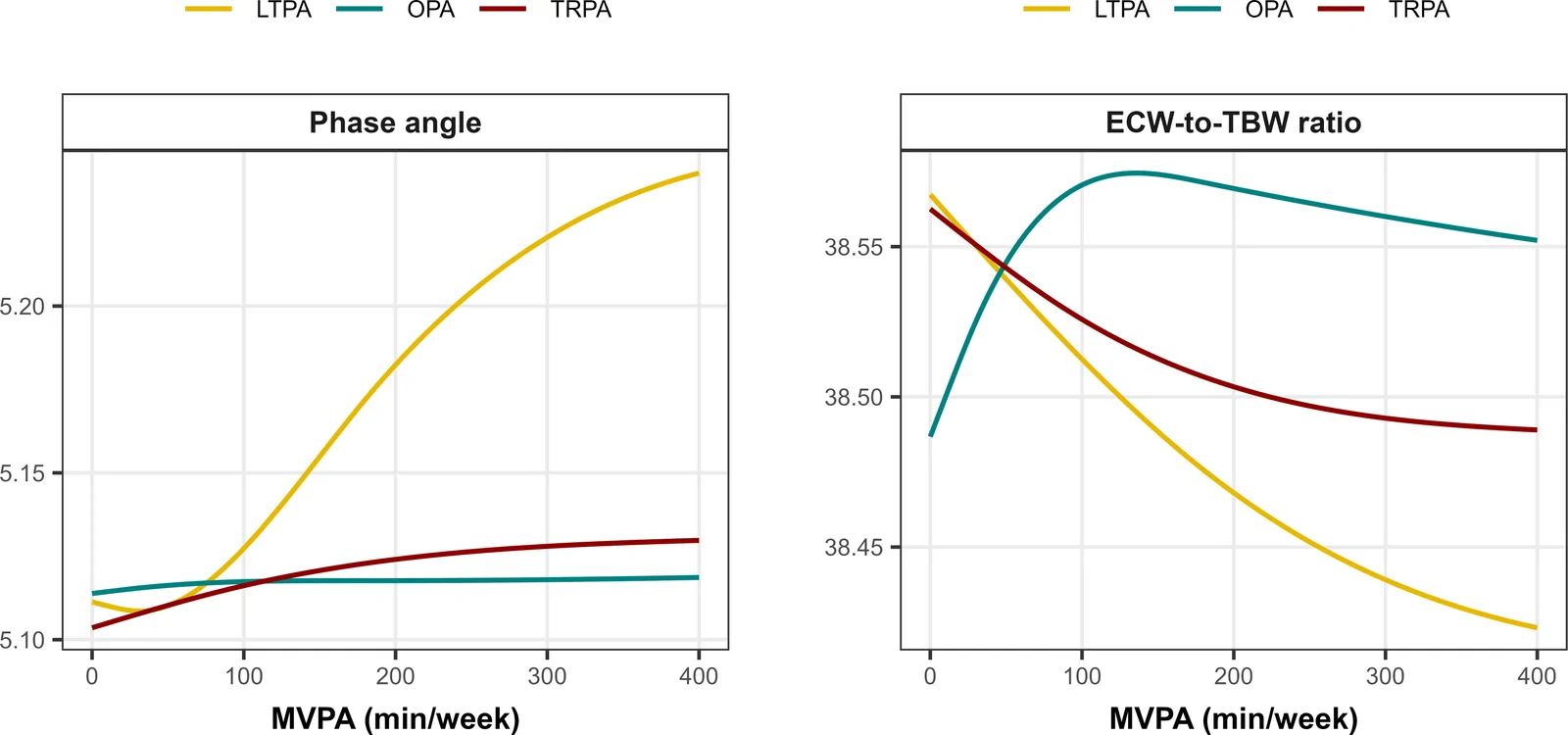
Nonlinear associations of leisure-time, occupational, and transport-related physical activity with phase angle and the extracellular water (ECW)–to–total body water (TBW) ratio, estimated using restricted cubic spline functions. The models were adjusted for sex, age, education, income, marital status, smoking status, alcohol consumption, hypertension, diabetes, chronic kidney disease, and BMI. LTPA: leisure-time physical activity; MVPA: moderate-to-vigorous physical activity; OPA: occupational physical activity; TRPA: transport-related physical activity.

## Discussion

### Principal Findings

This study investigated the domain-specific associations of PA with PhA and the ECW-to-TBW ratio in older adults. Total PA was positively associated with PhA and negatively associated with the ECW-to-TBW ratio in older adults. Among the PA domains, LTPA showed the most consistent associations with both PhA and the ECW-to-TBW ratio, whereas high levels of TRPA (≥300 min/week) were negatively associated only with the ECW-to-TBW ratio. Specifically, the nonlinear models demonstrated that the positive association between LTPA and PhA, as well as the negative association between LTPA and the ECW-to-TBW ratio, followed a largely linear pattern without a clear threshold. In contrast, OPA showed no clear associations with either PhA or the ECW-to-TBW ratio.

The results of this study are consistent with prior research demonstrating that PA is positively associated with PhA and negatively associated with the ECW-to-TBW ratio. Studies conducted in South Korea and Poland showed that self-reported PA was associated with a higher PhA among adults [19,29]. Furthermore, previous studies have consistently confirmed the positive association between objectively measured PA and PhA [20,30,31]. Evidence from a meta-analysis also supports the finding that PA is positively associated with PhA [32]. Conversely, previous research reported negative associations between PA levels and the ECW-to-TBW or ECW-to-ICW ratios [18,33]. A recent study using the doubly labeled water method found that PA levels were directly associated with PhA and inversely associated with the ECW-to-ICW ratio [34]. Building on this evidence, the present study provides novel insights by elucidating that the associations between PA and PhA and the ECW-to-TBW ratio vary substantially across PA domains.

It is hypothesized that complex physiological and biochemical mechanisms may underlie the associations between PA and both PhA and the ECW-to-TBW ratio. One of the biological pathways through which PA is linked to BIA-measured outcomes may be associated with the mitigation of systemic inflammation. Long-term engagement in PA was linked to lower levels of circulating inflammatory markers, including C-reactive protein, interleukin-6, and tumor necrosis factor α [35,36]. Chronic inflammation and oxidative stress can induce cellular damage, such as structural injury, membrane disruption, and apoptosis, thereby lowering PhA. Consequently, regular PA may be associated with higher PhA values [12]. Furthermore, PA promotes favorable changes in body composition by lowering body fat percentage and increasing skeletal muscle mass [37]. Such physiological adaptations to PA are also associated with a lower ECW-to-TBW ratio, reflecting improved intracellular-to-extracellular water balance [38].

Although this study found substantial associations between PA domains and PhA and the ECW-to-TBW ratio, the magnitudes of these associations were modest; for example, engaging in ≥300 min/week of LTPA was associated with a 0.13° higher PhA and a 0.2% lower ECW-to-TBW ratio compared with the 0 min/week. The modest effect sizes of the observed associations may be because BIA-derived profiles are influenced by a wide range of underlying demographic and health-related factors, including sex, age, and chronic conditions. Although these associations may appear modest at the individual level (ie, a 0.13° higher PhA and a 0.2% lower ECW-to-TBW ratio), they correspond to approximately 2 SDs of the distributions of PhA and the ECW-to-TBW ratio in our study population. Therefore, these differences may have meaningful implications at the population level.

The findings that the associations between PA and both PhA and the ECW-to-TBW ratio differed across PA domains warrant attention. These results align with the existing “PA paradox” hypothesis, which posits that the beneficial health effects of PA are primarily derived from LTPA [39]. A study of Korean adults reported that while LTPA was linked to lower high-sensitivity C-reactive protein levels, OPA was associated with higher levels [40]. Furthermore, a recent study of older adults in South Korea found that LTPA was positively associated with the skeletal muscle mass index, whereas OPA showed no significant association [41]. Although multiple factors may contribute to the differential associations of PA domains with health outcomes, one possible explanation is that LTPA generally involves the free and coordinated use of muscle groups throughout the body, whereas OPA often requires repetitive use of specific muscle groups [42]. Evidence from these studies suggests that LTPA is associated with beneficial physiological adaptations, which may explain why LTPA exhibited the most consistent associations with both PhA and the ECW-to-TBW ratio. Particularly for LTPA, individuals can freely determine the frequency and intensity of their activity. Therefore, those with better underlying health status may be more likely to engage in more frequent and vigorous LTPA, which may increase the potential for reverse causation and contribute to the stronger associations observed in the present study. Additionally, individuals with high levels of OPA may be more likely to have lower socioeconomic status and engage in occupations involving substantial physical demands and work-related stress, factors that may be associated with lower PhA values and higher ECW-to-TBW ratios. Such coexposure may have obscured the independent association between OPA and these BIA parameters. Furthermore, OPA was assessed using self-reported questionnaires rather than objective measures, which may not accurately capture the intensity, posture, and frequency of OPA. This limitation may have introduced measurement error and further attenuated the observed associations. Future longitudinal studies are needed to confirm the causal relationship.

The present study has several limitations. First, because of its cross-sectional design, the present study cannot establish the causal effects of PA on PhA and the ECW-to-TBW ratio. For instance, the possibility of reverse causation should be acknowledged; individuals in better physical condition, indicated by higher PhA and lower ECW-to-TBW ratios, may be more likely to engage in PA. This may be particularly relevant for LTPA, as individuals can freely determine the levels and intensity of PA. Therefore, the possibility of reverse causation may be greater for LTPA than for other PA domains and may have contributed to the stronger observed associations. Therefore, the temporal relationship between PA and both PhA and the ECW-to-TBW ratio should be clarified in future longitudinal studies. Second, while the K-GPAQ is a validated tool for evaluating PA among the Korean population and offers the advantage of differentiating LTPA, OPA, and TRPA, potential measurement errors arising from recall or social desirability bias should be recognized. A previous study reported that older adults tend to overreport their levels of PA when assessed using the GPAQ compared with accelerometer-based measurements [22]. Although self-reported and objectively measured PA were significantly correlated, the strength of this correlation was only modest, highlighting the inherent limitations and potential measurement error associated with questionnaire-based PA assessment [22]. Furthermore, the degree of measurement error may vary across PA domains, potentially resulting in differential misclassification and influencing the observed domain-specific associations. It should also be noted that, among older adults, the distinction between TRPA and LTPA may not be clear. Future studies would benefit from using objective measures, such as accelerometers, to improve accuracy. Third, this study did not account for several unmeasured confounders due to the lack of available data. For example, factors such as dietary intake, inflammatory biomarkers, and the use of medications that influence body composition and fluid status were not adjusted for in the regression models. Future in-depth analyses should incorporate these variables to elucidate the physiological mechanisms underlying the associations between PA and both PhA and the ECW-to-TBW ratio.

Despite these limitations, the study has some strengths. To the best of our knowledge, this is the first study to demonstrate domain-specific associations between PA and both PhA and the ECW-to-TBW ratio. Furthermore, the results were derived from a nationally representative sample of middle-aged and older adults, thereby ensuring the generalizability of the results.

### Conclusions

This study revealed that PA is differentially associated with PhA and the ECW-to-TBW ratio according to its domain in older adults in South Korea. LTPA demonstrated a notable positive association with PhA and a negative association with the ECW-to-TBW ratio, even at lower levels of activity. The negative association between TRPA and the ECW-to-TBW ratio was observed at higher levels (≥300 min/week), whereas OPA showed no clear associations with either PhA or the ECW-to-TBW ratio. Future research should use longitudinal study designs and objective measures of PA to establish the temporal sequence of the observed associations and improve the accuracy of PA assessment.

## Supplementary material

10.2196/90158 Multimedia Appendix 1 Sample characteristics, sensitivity analyses using imputed datasets, and domain-specific and continuous physical activity associations with phase angle and extracellular water–to–total body water ratio.

## Abbreviations

ECW: extracellular water
ICW: intracellular water
K-GPAQ: Korean version of the Global Physical Activity Questionnaire
KDCA: Korea Disease Control and Prevention Agency
KNHANES: Korea National Health and Nutrition Examination Survey
LTPA: leisure-time physical activity
MVPA: moderate to vigorous physical activity
OPA: occupational physical activity
PA: physical activity
PhA: phase angle
TBW: total body water
TRPA: transport-related physical activity
WHO: World Health Organization
