# Supplementary material for: Domain-Specific Associations of Physical Activity With Phase Angle and Extracellular Water–to–Total Body Water Ratio in Middle-Aged or Older Adults: Cross-Sectional Study
Source: JMIR Aging. 2026 Sep 28;9:e90158. doi: 10.2196/90158 (PMC13619118; doi:10.2196/90158)
Supplement: Supplementary file 1 [file aging-v9-e90158-s001.docx]

**Table S1.** Characteristics of the included and excluded samples

|  | **Included sample** | **Excluded sample** | **P value** |
| --- | --- | --- | --- |
|  | **N = 5,211** | **N = 531** |  |
| Sex |  |  | 0.106 |
| Male | 2,256 (43.3%) | 210 (39.5%) |  |
| Female | 2,955 (56.7%) | 321 (60.5%) |  |
| Age (years) |  |  | < 0.001 |
| Mean (SD) | 64.5 (8.7) | 69.4 (9.5) |  |
| Education level |  |  | < 0.001 |
| Middle school or below | 2,111 (40.5%) | 192 (58.2%) |  |
| High school | 1,770 (34.0%) | 83 (25.2%) |  |
| College or above | 1,330 (25.5%) | 55 (16.7%) |  |
| Missing values | 0 | 201 |  |
| Income level |  |  | < 0.001 |
| Lowest | 1,362 (26.1%) | 231 (44.8%) |  |
| Low | 1,344 (25.8%) | 139 (26.9%) |  |
| High | 1,175 (22.5%) | 74 (14.3%) |  |
| Highest | 1,330 (25.5%) | 72 (14.0%) |  |
| Missing values | 0 | 15 |  |
| Marital status |  |  | < 0.001 |
| Married | 3,979 (76.4%) | 331 (62.5%) |  |
| Unmarried | 149 (2.9%) | 12 (2.3%) |  |
| Others | 1,083 (20.8%) | 187 (35.3%) |  |
| Missing values | 0 | 1 |  |
| Smoking |  |  | 0.809 |
| No | 4,503 (86.4%) | 377 (85.9%) |  |
| Yes | 708 (13.6%) | 62 (14.1%) |  |
| Missing values | 0 | 92 |  |
| Alcohol |  |  | 0.003 |
| No | 2,920 (56.0%) | 285 (63.5%) |  |
| Yes | 2,291 (44.0%) | 164 (36.5%) |  |
| Missing values | 0 | 82 |  |
| Hypertension |  |  | < 0.001 |
| No | 3,176 (60.9%) | 260 (49.1%) |  |
| Yes | 2,035 (39.1%) | 269 (50.9%) |  |
| Missing values | 0 | 2 |  |
| Diabetes |  |  | < 0.001 |
| No | 4,346 (83.4%) | 401 (75.8%) |  |
| Yes | 865 (16.6%) | 128 (24.2%) |  |
| Missing values | 0 | 2 |  |
| CKD |  |  | 0.796 |
| No | 5,049 (96.9%) | 152 (96.2%) |  |
| Yes | 162 (3.1%) | 6 (3.8%) |  |
| Missing values | 0 | 373 |  |
| Body mass index (kg/m^2^) |  |  | 0.575 |
| Means (SD) | 24.1 (3.3) | 24.0 (3.4) |  |
| Missing values | 0 | 77 |  |
| Phase angle (°) |  |  | < 0.001 |
| Mean (SD) | 5.1 (0.7) | 4.8 (0.8) |  |
| ECW/TBW ratio (%) |  |  | < 0.001 |
| Mean (SD) | 38.6 (0.8) | 39.0 (1.0) |  |

Chi-squared test for categorized variables and t-test for continuous variables.

**Table S2** Associations of total physical activity with the phase angle and ECW/TBW ratio based on the imputed datasets (n = 5,742)

|  | **Phase angle (°)** | | **ECW/TBW ratio (%)** | |
| --- | --- | --- | --- | --- |
|  | **Crude model** | **Adjusted model** | **Crude model** | **Adjusted model** |
|  | **β (95% CI)** | **β (95% CI)** | **β (95% CI)** | **β (95% CI)** |
| **Total PA (MVPA)** |  |  |  |  |
| 0 min/week | Reference | Reference | Reference | Reference |
| 1­–149 min/week | -0.02 (-0.08, 0.05) | 0.02 (-0.01, 0.06) | -0.04 (-0.11, 0.02) | -0.04 (-0.09, -0.00) |
| 150–299 min/week | 0.10 (0.03, 0.18) | 0.04 (-0.01, 0.09) | -0.21 (-0.29, -0.13) | -0.09 (-0.14, -0.03) |
| ≥300 min/week | 0.24 (0.17, 0.31) | 0.08 (0.04, 0.12) | -0.30 (-0.37, -0.23) | -0.11 (-0.15, -0.06) |

PA, physical activity; MVPA, moderate-to-vigorous physical activity; ECW, extracellular water; TBW, total body water; CI, confidence interval

**Table S3** Associations of leisure-time, occupational, and transport-related physical activity with the phase angle and ECW/TBW ratio based on the imputed datasets (n = 5,742)

|  | **Phase angle (°)** | | **ECW/TBW ratio (%)** | |
| --- | --- | --- | --- | --- |
|  | **Crude model** | **Adjusted model** | **Crude model** | **Adjusted model** |
|  | **β (95% CI)** | **β (95% CI)** | **β (95% CI)** | **β (95% CI)** |
| **LTPA (MVPA)** |  |  |  |  |
| 0 min/week | Reference | Reference | Reference | Reference |
| 1­–149 min/week | 0.22 (0.15, 0.29) | 0.01 (-0.03, 0.06) | -0.31 (-0.37, -0.24) | -0.05 (-0.11, 0.00) |
| 150–299 min/week | 0.26 (0.18, 0.35) | 0.07 (0.01, 0.13) | -0.35 (-0.45, -0.25) | -0.10 (-0.19, -0.02) |
| ≥300 min/week | 0.45 (0.35, 0.55) | 0.13 (0.06, 0.20) | -0.47 (-0.56, -0.37) | -0.14 (-0.22, -0.07) |
| **OPA (MVPA)** |  |  |  |  |
| 0 min/week | Reference | Reference | Reference | Reference |
| 1­–149 min/week | 0.14 (0.01, 0.26) | 0.05 (-0.04, 0.13) | -0.05 (-0.17, 0.08) | 0.00 (-0.09, 0.10) |
| 150–299 min/week | 0.04 (-0.18, 0.25) | -0.08 (-0.20, 0.04) | 0.02 (-0.21, 0.25) | 0.12 (-0.03, 0.27) |
| ≥300 min/week | 0.31 (0.21, 0.42) | 0.02 (-0.05, 0.09) | -0.22 (-0.32, -0.11) | 0.06 (-0.02, 0.15) |
| **TRPA (MVPA)** |  |  |  |  |
| 0 min/week | Reference | Reference | Reference | Reference |
| 1­–149 min/week | -0.13 (-0.19, -0.08) | 0.00 (-0.03, 0.04) | 0.07 (0.01, 0.12) | -0.03 (-0.07, 0.01) |
| 150–299 min/week | -0.05 (-0.12, 0.02) | 0.02 (-0.03, 0.07) | -0.03 (-0.10, 0.04) | -0.06 (-0.11, -0.00) |
| ≥300 min/week | -0.03 (-0.11, 0.06) | 0.03 (-0.03, 0.09) | -0.03 (-0.11, 0.06) | -0.08 (-0.14, -0.02) |

LTPA, leisure-time physical activity; OPA, occupational physical activity; TRPA, transport-related physical activity; MVPA, moderate-to-vigorous physical activity; ECW, extracellular water; TBW, total body water; CI, confidence interval

**Table S4** Associations of leisure-time, occupational, and transport-related physical activity with the phase angle and ECW/TBW ratio using the continuous PA amount

|  | **Phase angle (°)** | | **ECW/TBW ratio (%)** | |
| --- | --- | --- | --- | --- |
|  | **Crude model** | **Adjusted model** | **Crude model** | **Adjusted model** |
|  | **β (95% CI)** | **β (95% CI)** | **β (95% CI)** | **β (95% CI)** |
| **Model 1** |  |  |  |  |
| **Total PA (MVPA)** |  |  |  |  |
| Unit: 150-min increment | 0.05 (0.04, 0.06) | 0.01 (0.00, 0.02) | -0.05 (-0.06, -0.04) | -0.01 (-0.02, -0.01) |
| **Model 2 (domain-specific)** |  |  |  |  |
| **LTPA (MVPA)** |  |  |  |  |
| Unit: 150-min increment | 0.11 (0.08, 0.13) | 0.03 (0.01, 0.05) | -0.11 (-0.14, -0.09) | -0.03 (-0.05, -0.01) |
| **OPA (MVPA)** |  |  |  |  |
| Unit: 150-min increment | 0.04 (0.03, 0.06) | 0.00 (-0.01, 0.01) | -0.03 (-0.04, -0.02) | 0.01 (-0.01, 0.02) |
| **TRPA (MVPA)** |  |  |  |  |
| Unit: 150-min increment | -0.00 (-0.02, 0.02) | 0.01 (-0.01, 0.02) | -0.01 (-0.03, 0.01) | -0.02 (-0.04, -0.01) |

LTPA, leisure-time physical activity; OPA, occupational physical activity; TRPA, transport-related physical activity; MVPA, moderate-to-vigorous physical activity; ECW, extracellular water; TBW, total body water; CI, confidence interval
